# A prophylactic effect of aluminium-based adjuvants against respiratory viruses via priming local innate immunity

**DOI:** 10.1080/22221751.2022.2050951

**Published:** 2022-03-28

**Authors:** Xin Wang, Xiaochen Yin, Boya Zhang, Chenfeng Liu, Yahua Lin, Xiaofen Huang, Yufang Li, Chenguang Shen, Weibin Zheng, Guofeng Fu, Junyu Chen, Yanling Wen, Wei Zhang, Bo-Sheng Pan, Mujin Fang, Zizheng Zheng, Zheng Zhang, Quan Yuan, Guo Fu, Shaowei Li, Jun Zhang, Yixin Chen, Ningshao Xia, Qinjian Zhao

**Affiliations:** a State Key Laboratory of Molecular Vaccinology and Molecular Diagnostics, National Institute of Diagnostics and Vaccine Development in Infectious Diseases, School of Public Health, Xiamen University, Xiamen, People’s Republic of China; b Institute for Hepatology, National Clinical Research Center for Infectious Disease, Shenzhen Third People’s Hospital; The Second Affiliated Hospital, School of Medicine, Southern University of Science and Technology, Shenzhen, People’s Republic of China; c School of Life Science, Xiamen University, Xiamen, People’s Republic of China; d Department of Cell Biology, School of Life Science, Anhui Medical University, Hefei, People’s Republic of China; e College of Pharmacy, Chongqing Medical University, Chongqing, People’s Republic of China; f Reverie Labs, Cambridge, MA, USA

**Keywords:** Prophylactic, influenza, respiratory syncytial virus, alveolar macrophage, interferon

## Abstract

Infection caused by respiratory viruses can lead to a severe respiratory disease and even death. Vaccination is the most effective way to prevent the disease, but it cannot be quickly applied when facing an emerging infectious disease. Here, we demonstrated that immunization with an aluminium-zinc hybrid particulate adjuvant (FH-001) alone, bearing great resemblance in morphology with commonly used aluminium-based adjuvants in vaccines, could quickly induce mice to generate a broadly protective immune response to resist the lethal challenge of influenza B viruses. Furthermore, a multi-omics-based analysis revealed that the alveolar macrophage and type I interferon pathway, rather than adaptive immunity and type II interferon pathway, were essential for the observed prophylactic effect of FH-001. More importantly, a similar protective effect was observed against influenza A virus strain A/Shanghai/02/2013(H7N9), A/California/04/2009(H1N1) and respiratory syncytial virus. Therefore, we introduced here a new and promising strategy that can be quickly applied during the outbreak of emerging respiratory viruses.

## Introduction

Respiratory viruses are important pathogens that cause human respiratory diseases. These infections can manifest mostly as fever and cough, but in some cases, these infections can lead to moderate to severe pneumonia and acute respiratory distress syndrome in severe cases. For instance, the influenza H1N1 in 1918 [1], the influenza H2N2 in 1957, the Severe Acute Respiratory Syndrome Coronavirus (SARS-CoV) in 2002 [2], and the ongoing novel coronavirus (SARS-CoV-2) that emerged at the end of 2019 [3–7] significantly changed the socio-economical life worldwide.

Vaccination and broad-spectrum neutralizing antibodies [8–10] are the most effective ways to prevent and treat the infection of respiratory viruses and also to prevent the occurrence of severe disease. The rapid mutation capabilities of the respiratory RNA viruses [11] can render the vaccines or therapeutic antibodies ineffective due to the long development cycles of vaccines or drugs not matching up with the speed of viral mutations. For example, the constituent strains of influenza vaccines need to be predicted every year based on the worldwide epidemic situation [12]. A retrospective study in 2014 showed that, between 1999 and 2012, the strains of influenza B virus used in the vaccine were mismatched seven times [13]. More recently, the variants of SARS-CoV-2 such as Delta and Omicron exhibited potent capabilities for immune evasion [14–16]. The research and development of vaccines could not respond to the emerging outbreaks on time. Notably, the outbreak of the new coronavirus SARS-CoV-2 has already had a great impact on society and the economy [17,18]. Even though numerous vaccines were approved for Coronavirus disease 2019 (COVID-19), the emerging mutated SARS-CoV-2 variants reduced the effectiveness of the vaccines. Therefore, effective prophylactic drugs would be greatly beneficial for disease prevention and control.

Most vaccines, particularly the inactivated virus- and recombinant protein-based vaccines, contain adjuvants for delivery and immunopotentiation. In 1926, *Glenny* et al. discovered that the immunogenicity of diphtheria toxin was significantly enhanced upon precipitation with potassium aluminium sulphate [19]. Even after more than 90 years, the aluminium-containing adjuvant is still the most widely used human vaccine adjuvant [20]. Interestingly, *Wang* et al. discovered that intraperitoneal injection with aluminium adjuvant alone can activate CD8^+^ T cells, thereby killing tumour cells that were subcutaneously inoculated in mouse and significantly reducing the tumour volume in a non-specific way [21]. Besides the adjuvant, some studies reported that granulocyte/macrophage colony-stimulating factor in the lungs also increase resistance to influenza [22]. Furthermore, *Proud* et al. demonstrated that intranasally administered TLR2/6 agonist in a ferret model induced a prophylactic effect against SARS-CoV-2 [23]. More commonly, a nasal spray of type I interferon protects human beings from respiratory viruses [24]. These results indicated that prophylactic stimulators have great potential to protect the host against various diseases. However, the toll-like receptor (TLR) agonists or cytokines generally provide a short protection time. Therefore, a new stimulator with a relatively long-term protection effect and safety is important, such as the aluminium-based adjuvant, which has been widely used in human beings for over 90 years.

In this study, we used a well-established mouse-adapted influenza B virus-based mouse challenge model to investigate the prophylactic effect of an optimized aluminium-based adjuvant (namely FH-001, with zinc as a critical component). We found that the FH-001 could provide a relatively long-term protection effect against influenza B viruses, influenza A viruses, and respiratory syncytial virus. Mechanically, the existence of alveolar macrophage and type I interferon related pathway were shown to be important for the prophylactic effect.

## Materials and methods

### Mice

All animals used in this study were 6- to 8-week-old female BALB/c mice (LING CHANG, Inc., Shanghai, China). The CB17/Icr-Prkdc^scid^/IcrlcoCrl (C.B-17 SCID, Strain code: 404) and NOD.CB17-Prkdc^scid^/NcrCrl (NOD-SCID, Strain code: 406) were purchased from The Beijing Vital River Laboratory Animal Technology Co. Ltd. The animal protocols were in accordance with the Institutional Animal Care and Use Committee guidelines and were approved by the Ethics Committee of Xiamen University Laboratory Animal Center.

### Preparation of Al-001 and FH-001

The Al-001 and FH-001 were prepared in-house based on established procedures [25–28]. Specifically, these amorphous particulate adjuvants containing aluminium were prepared by co-precipitation via in-line mixing or in a batch mode. The Al concentration in both Al-001 and FH-001 was 420 μg/mL. In addition, there was 380 μg /mL Zn in the newly developed FH-001 where Zn was doped in the commonly used Al-based adjuvants. The size, point-of-zero-charge, and protein adsorption capacity were evaluated.

### Prophylactic efficacy studies in mice

For most prophylactic assays, female BALB/c mice aged 6–8 weeks were intranasally administered with 100 μL FH-001/Al-001 (containing 3.11 × 10^−4^ mmol aluminium) or 100 μL physiological saline. As reported previously, a dose volume of 50 μL or more could be efficiently delivered into the lungs via intranasal instillation [29,30]. Seven days later, the mice were deeply anesthetized with isoflurane and oxygen and challenged intranasally with 25-fold LD_50_ of B/Florida/4/2006 (3.50 × 10^3^ PFU) or A/Shanghai/02/2013(H7N9) (3.50 × 10^4^ PFU) or A/California/04/2009(H1N1) (1.75 × 10^3^ PFU). In addition, 2.00 × 10^5^ PFU of RSV A2 were used to challenge the mice. For the liposome-based depletion assay, the mice received 100 μL C.L.L. (LIPOSOMA BV, SKU: CP-005-005) or 100 μL control liposome (LIPOSOMA BV, SKU: CP-005-005) at 2, 5, 8, and 10 days before infection. For IFNAR-1 blocking studies, mice were administered MAR1-5A3 (25 mg/kg) or control isotype antibody (25 mg/kg) intranasally five times at 2, 4, 6, 7, and 8 days before viral challenge. For IFN-γ neutralizing studies, mice were administered XMG1.2 (17.5 mg/kg) or control isotype antibody (17.5 mg/kg) intranasally four times at 2, 4, 6, and 7 days before viral challenge. The animals were observed daily for mortality and morbidity, and body weight was measured for 14 days after infection. Animals that lost more than 25% of their initial body weight were euthanized in accordance with our animal ethics protocol.

### Flow cytometry analysis

mAbs against mouse CD3 (FITC), CD4 (APC), CD8 (APC/Cy7), CD11b (eF450), CD11c (APC), Ly-6G (Percy/Cy5.5), Ly-6C (PE/Cy7), Gr1.1 (PerCP/cy5.5), F4/80 (APC-Cy7), CD19 (PE), and B220 (FITC) were purchased from BD PharMingen (San Diego, CA). Cell suspensions were treated with Mouse Fc Block^TM^ (BD PharMingen, CA) on ice for 15 min before staining with various combinations of mAbs for 30 min on ice. Cells were washed twice with PBS with 1% FCS before analysis on a FACS Calibur (BD Biosciences). A total of 80,000 events were acquired for each lung sample. The data were analysed by FlowJo (Tree Star Inc., Ashland, OR).

### Haematoxylin–eosin staining

The prophylactic agents or physiological saline-treated mice were killed on the 12th day after the influenza virus challenge or on the fifth day after the RSV challenge. Then, the lungs were collected and fixed by 4% formalin for 24 h. HE staining was conducted according to routine protocols. Briefly, after deparaffinization and rehydration, 5-μm longitudinal sections were stained with haematoxylin solution for 5 min, followed by five dips in 1% acid ethanol (1% HCl in 70% ethanol), and then rinsed in distilled water. Then, the sections were stained with eosin solution for 3 min, followed by dehydration with graded alcohol and clearing in xylene. The mounted slides were then examined and photographed using an Olympus BX51 fluorescence microscope (Tokyo, Japan).

### Evaluation of total antibodies and virus-specific antibodies

Enzyme-linked immunosorbent assay (ELISA) as described before [31,32] was used to test the antibody response of FH-001 treated mice, lung homogenate (centrifuge to obtain supernatant and discard pellet) and serum were collected from mice at seventh day after intranasally administration of 10-fold diluted FH-001 or physiological saline.

For the evaluation of total antibodies, 100 ng/well Goat-anti-Mouse (GAM) antibodies were coated in the 96-well ELISA plate as capture antibody, and GAM-HRP were used 1:5000 in dilution buffer (PBS containing 0.5% casein, 2% gelatin and 0.1% Proclin) as detection antibody. For specific classes of immunoglobulin, GAM is also used as capture antibody, while the detection antibody was changed to anti-IgG_1_-HRP (1: 5000), or anti-IgG_2a_-HRP (1: 5000), or anti-IgG_2b_-HRP (1: 5000), or anti-IgG_3_-HRP (1: 5000), or anti-IgA-HRP (1: 5000), or anti-IgM-HRP (1: 5000). The dilution folds of lung homogenates or serum samples were pre-tested to make the baseline of OD_450_ value of each immunoglobulin classes to be between 0.5 and 2.0.

For the evaluation of virus-specific antibodies, 100 ng/well of formalin-inactivated FL/2006 were coated in the 96-well ELISA plate as capture antigen, the lung homogenates or serums were 10-fold diluted and the detection antibody GAM-HRP were 5000-fold diluted in dilution buffer. For specific classes of immunoglobulin, formalin-inactivated FL/2006 is used as capture antigen, while the detection antibody was changed to anti-IgG_1_-HRP (1: 1000), or anti-IgG_2a_-HRP (1: 1000), or anti-IgG_2b_-HRP (1: 1000), or anti-IgG_3_-HRP (1: 1000), or anti-IgA-HRP (1: 1000), or anti-IgM-HRP (1: 1000), all capture antibody and detection antibodies are purchased from Invitrogen (Thermo Fisher Scientific).

### Transmission electron microscopes (TEM)

The FH-001 and Al-001 were diluted with respective buffers in the ratio 1:50. The sections were expanded using chloroform vapour and caught on 200 mesh, thin bar 3.05-mm copper grids (Athene, UK). The images were obtained using a Tecnai™ G2 Spirit TEM (FEI, Irchel, Zurich) transmission electron microscope.

### HA test

For the HA test, 50 µL of 0.5% TRBCs was added to 50 µL of 2-fold serially diluted cell culture supernatant, and the mixture was incubated at room temperature for 1 h. The assay was performed in quadruplicate. The lowest concentration of virus that was bound to TRBCs was designated as the HA titer [8].

### Statistical analysis

The two-tailed unpaired Student’s *t* test (Prism version 8.0.1, GraphPad Software, San Diego, CA) was used to compare differences between the abundance of cell types for all cell clusters in different groups. A *p*-value < 0.05 was considered statistically significant.

## Results

### The prophylactic effect of Al-001 and FH-001 against influenza B virus

**Figure 1.**
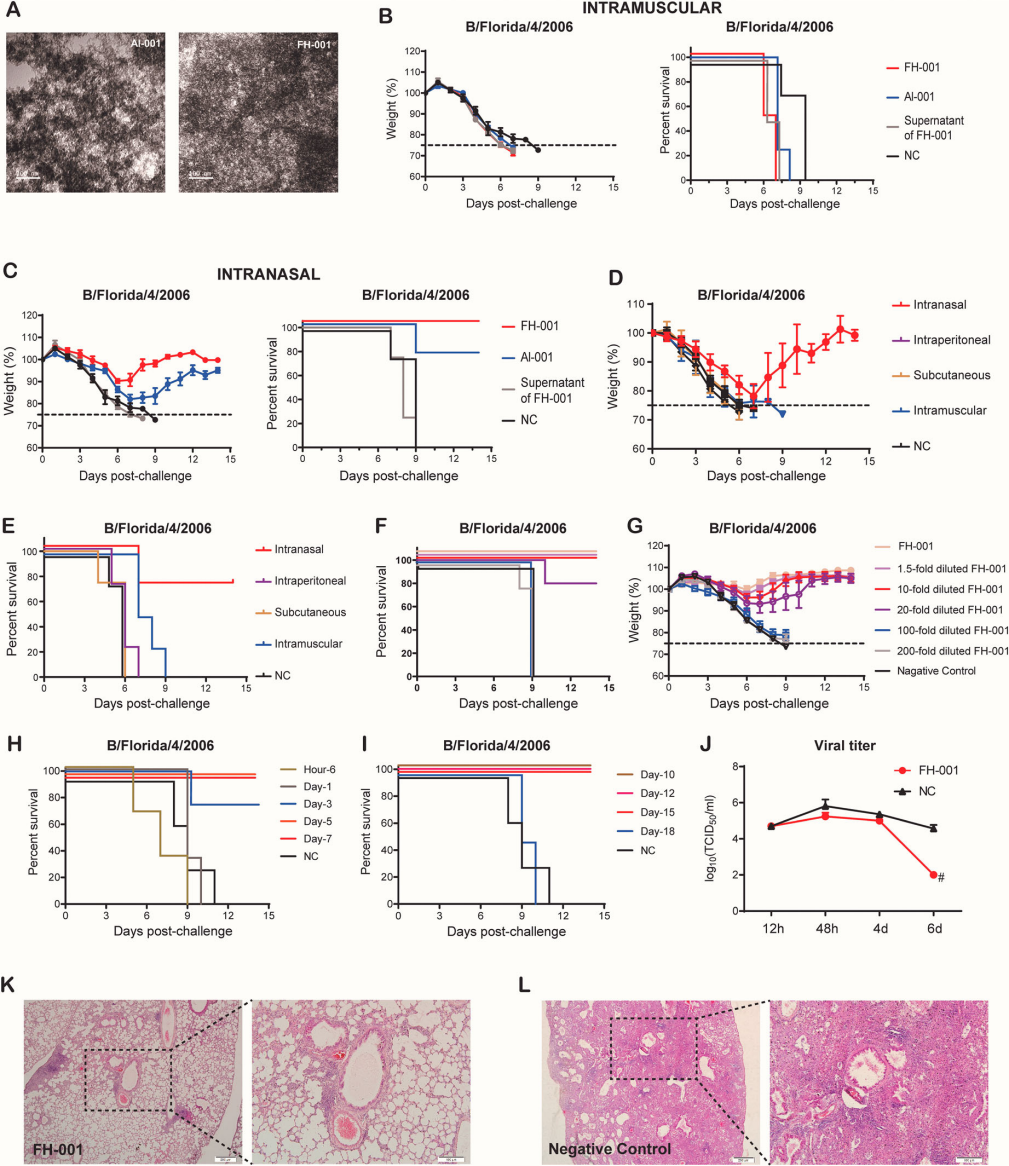
Prophylactic effect of aluminium-based adjuvants FH-001 and Al-001. (A) Transmission electron microscopy of FH-001 and Al-001. Scale bar, 100 nm. (B) Survival rate and weight change of BALB/c mice after intramuscular administration of FH-001 and lethal challenge by B/Florida/4/2006 (Short as “FL/2006”). The BALB/c mice that were intramuscularly administered with physiological saline were challenged by FL/2006 and used as the negative control (NC) group, *n* = 4 mice per group. (C) Survival rate and weight change of BALB/c mice after intranasal administration of FH-001 and lethal challenge by FL/2006. The BALB/c mice that were intranasally administered with physiological saline were challenged by FL/2006 and used as NC group, *n* = 4 mice per group. (D-E) Survival rate and weight change of BALB/c mice after administration of FH-001 via different routes and lethal challenge by FL/2006, *n* = 4 mice per group. (F) Survival rate of 1- to 200-fold diluted FH-001 treated BALB/c mice after a lethal challenge of FL/2006. The BALB/c mice that were intranasally administered with physiological saline were challenged by FL/2006 and used as NC group. Five BALB/c mice were used in each FH-001 treated group, four mice were used in NC group. (G) Fourteen days weight change of 1- to 200-fold diluted FH-001 treated mice after FL/2006 challenge. (H–I) The survival rate of 10-fold diluted FH-001 treated mice (treated for different durations) after the challenge of FL/2006 (*n* = 4 mice per group). (J) Viral titer of FL/2006 in lungs from two groups (FH-001-treated mice or physiological saline-treated mice) at different time points after the challenge of the FL/2006. (K–L) Haematoxylin-eosin staining of lung tissues from FH-001 or 0.9% saline-treated mice after the challenge of FL/2006.

Al-001 and FH-001 are aluminium-based amorphous adjuvants (Figure 1(A)) that were produced in-house. To investigate the prophylactic effects of Al-001 and FH-001, we first pretreated BALB/c mice with the adjuvants alone or physiological saline (0.9% w/v) intramuscularly or intranasally. A lethal dose [9] of B/Florida/4/2006 (short as “FL/2006”) [9] was then used to challenge the BALB/c mice 1 week after adjuvant treatment. At the ninth day post-challenge, 100% of the BALB/c mice from the intramuscular group were dead (Figure 1(B)), while 75% and 100% of the BALB/c mice intranasally pretreated with Al-001 or FH-001, respectively, survived (Figure 1(C)). The weight loss of BALB/c mice from the FH-001 group ranged from 7.7% to 15%, while for the Al-001 group, the changes were 16%–22% (Figure 1(C)), suggesting that the prophylactic effect of FH-001 is greater than the effect of Al-001. Therefore, the FH-001 was chosen to determine whether the prophylactic effect of FH-001 is provided by the intranasal route.

We pretreated BALB/c mice with FH-001 through four different modes of administration: intranasal, intraperitoneal, subcutaneous, or intramuscular. After the lethal challenge of FL/2006, BALB/c mice from the “intranasal” group survived (Figure 1(D–E)), while the other BALB/c mice were all dead before the ninth day (Figure 1(D–E)). Therefore, intranasal administration route was used in the following studies. Subsequently, a series of diluted FH-001 preparations (i.e. 1.5-fold, 10-fold, 20-fold, 100-fold, and 200-fold diluted with saline) were intranasally administrated to mice for assessing the dose–effect relationship. After FL/2006 challenge, the BALB/c mice that intranasally pretreated with 1.5–10-fold diluted FH-001 were all survived (Figure 1(F–G)), and those BALB/c mice pretreated with 20-fold diluted FH-001 only exhibit 80% survival rate (Figure 1(F–G)), while the 100 or 200-fold diluted FH-001 showed no protection against FL/2006 (Figure 1(F–G)). A similar result was observed in Al-001 (Figure S1A–S1C). Considering that the weights of BALB/c mice were decreased after the FH-001 administration (Figure S1D), 10-fold diluted FH-001 was used for the following analysis to minimize the adverse effect and maximize the prophylactic effect.

Unlike vaccines, the protective effect of FH-001 or Al-001 was observed without the existence of any specific antigen. To investigate the duration of the protective effect, BALB/c mice were pretreated with FH-001 for different days, which ranged from 6 h to 18 days. After a lethal challenge of FL/2006, 75%–100% of the BALB/c mice that intranasally pretreated with FH-001 for 3–15 days were survived (Figure 1(D–E), Figure S1E–S1F), While the mice pretreated with FH-001 for 6 h, 1 day, or 18 days were all dead (Figure 1(D–E), Figure S1E–S1F).

To figure out if the pretreatment of FH-001 could significantly decrease the proliferation of FL/2006, the virus titers between FH-001 pretreated BALB/c mice and physiological saline pretreated BALB/c mice were comparatively analysed. The median tissue culture infectious dose (TCID_50_) was similar between the FH-001 group and the NC group at the beginning; however, the virus titers obtained from the FH-001 group were significantly decreased (TCID_50 _< 100/mL) at the sixth day after FL/2006 challenge (Figure 1(F)), while the TCID_50_ values were still over 10,000/mL in the negative control group (Figure 1(F)). Further haematoxylin–eosin staining revealed that the mouse lungs from BALB/c mice in the FH-001 group were relatively intact. The alveolar structure was clear, and inflammation and inflammatory cell infiltration only appeared in a few local areas. There was no obvious substantive and severe inflammation compared to the negative control (Figure 1(G–H), Figure S1G).

In summary, these results revealed that intranasal administration of Al-001 and FH-001 could elicit a prophylactic effect against the lethal challenge of influenza B virus FL/2006 for several weeks.

### The adaptive immune response is dispensable for the prophylactic effect of FH-001

**Figure 2.**
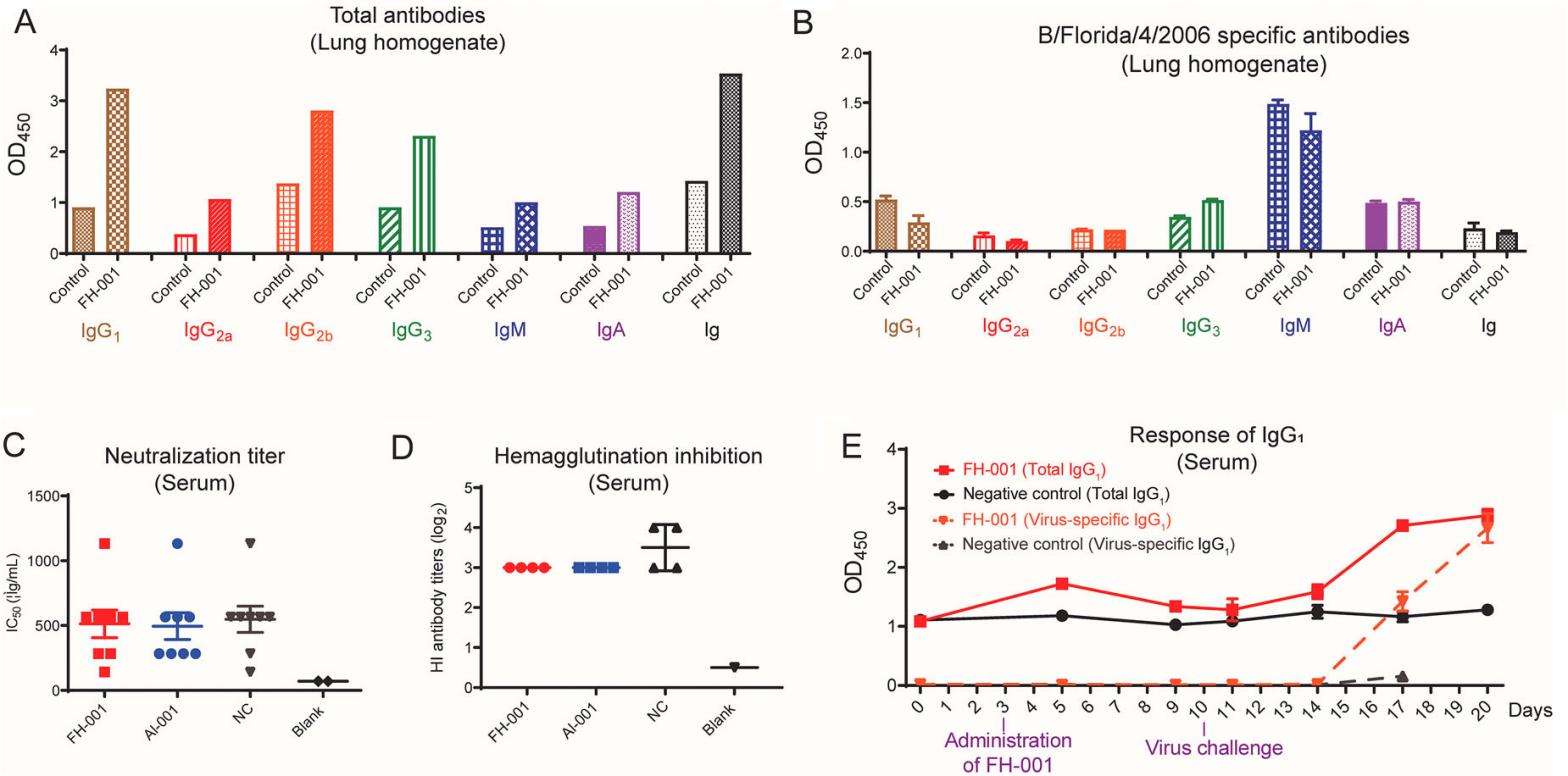
Antibody response after intranasal administration of FH-001. (A) Total antibody (non-specific antibody) response from lung homogenates of FH-001 or supernatant of FH-001 treated mice. (B) FL/2006 specific antibody response from lung homogenates in FH-001 or supernatant of FH-001 treated mice. (C) Neutralization titer to FL/2006. Serum of FH-001, Al-001, or physiological saline-treated mice were tested. The microwells added with PBS rather than serum were defined as blank. (D) Hemagglutination inhibition titer to FL/2006. Serum of FH-001, Al-001, or physiological saline-treated mice were tested. The microwells added with PBS rather than serum were defined as blank. (E) Continuous total IgG_1_ titer and FL/2006 specific IgG_1_ titer in serums from FH-001 treated and FL/2006 challenged mice.

To understand if adaptive immunity is a key factor for this prophylactic effect, the antibody titer at 7 days after FH-001 or Al-001 intranasal administration was tested. The total antibody from lung homogenate were increased in FH-001 treated BALB/c mice (Figure 2(A)), whereas no differences were found for the FL/2006 specific antibody titer (evaluated by inactivated FL/2006 coated ELISA) among FH-001 and physiological saline-treated BALB/c mice (Figure 2(B)). Furthermore, the FL/2006 specific antibody, neutralization titer and hemagglutination inhibition titer in serum of FH-001 treated mice were also not significantly change e(Figure 2(C, D), Figure S2A). After the virus challenge, the FH-001-treated BALB/c mice produced FL/2006-specific IgG1 antibody quicker in serum than the negative control BALB/c mice (Figure 2(E)), but not the IgA, IgM, or other antibody isotypes (Figure S2B–S2G). Consistently, the proportion and number of B220^+^ B cells were increased in FH-001-treated BALB/c mice compared to the NC group (Figure S2H–S2I). In addition, the number of CD8^+^ T cells was also increased (Figure S2J).

**Figure 3.**
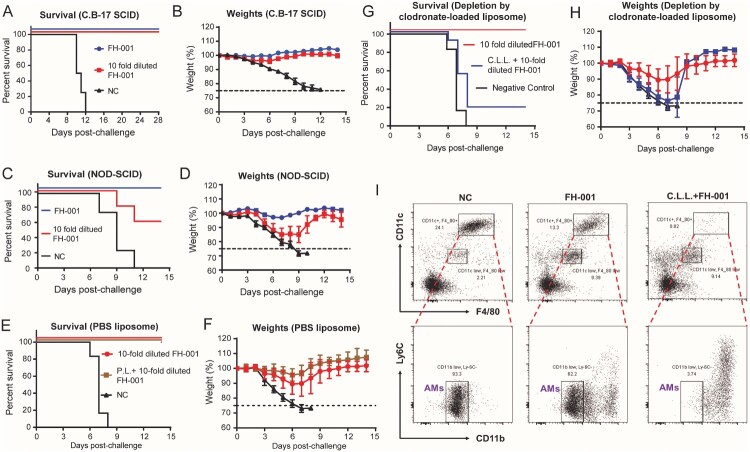
Alveolar macrophage, rather than adaptive immunity, is essential for the prophylactic effect of FH-001. (A) Survival rate of FH-001 or 10-fold diluted FH-001 treated C.B-17 SCID mice after FL/2006 challenge. The C.B-17 SCID mice that were intranasally administered with physiological saline were used as a negative control (*n* = 10 mice per FH-001 treated groups, *n* = 4 mice in negative control group). (B) Weight change of FH-001 or 10-fold diluted FH-001 treated C.B-17 SCID mice after FL/2006 challenge (*n* = 10 mice per FH-001 treated groups, *n* = 4 mice in negative control group). (C) Survival rate of FH-001 or 10-fold diluted FH-001 treated NOD-SCID mice after FL/2006 challenge. The NOD-SCID mice that were intranasally administered with physiological saline were challenged by FL/2006 and used as a negative control (*n* = 5 mice per FH-001 treated groups, *n* = 4 mice in negative control group). (D) Weight change of FH-001 or 10-fold diluted FH-001 treated NOD-SCID mice after FL/2006 challenge. (*n* = 5 mice per FH-001 treated groups, *n* = 4 mice in negative control group). (E–F) Prophylactic effect of 10-fold diluted FH-001 on control PBS liposome (P.L.) treated BALB/c mice after FL/2006 challenge. The survival rates are shown in Figure 3E; the weight changes are shown in Figure 3F (*n* = 8 mice in FH-001 and P.L. + FH-001 treated groups, *n* = 6 mice in negative control group). (G–H) Prophylactic effect of 10-fold diluted FH-001 on C.L.L. treated BALB/c mice after FL/2006 challenge (*n* = 8 mice in FH-001 group, *n* = 11 mice in C.L.L. + FH-001 group, *n* = 6 mice in negative control group). (I) Flow cytometry results of CD11b^low^ CD11c^+^ F4/80^+^ alveolar macrophage in the bronchoalveolar lavage fluid (BALF) of physiological saline, 10-fold diluted FH-001, or 10-fold diluted FH-001 + C.L.L. treated mice. The BALF was collected on day 7 after intranasal administration of each formulation but without viral challenge.

To verify the role of B cells and T cells in the FH-001-stimulated prophylactic effect, an immunodeficient C.B-17 SCID model was used. Flow cytometry analysis showed no leaky phenomenon in C.B-17 SCID (Figure S3A–S3B). After the lethal FL/2006 challenge, all FH-001 or 10-fold diluted FH-001 pretreated C.B-17 SCID mice survived (Figure 3(A–B), Figure S3C–S3D), while all C.B-17 SCID mice of the NC group died. Therefore, FH-001 pretreatment via intranasal administration could promote the generation of virus-specific IgG_1_ antibody, but the adaptive immunity is dispensable for the prophylactic effect of FH-001.

### Alveolar macrophage is critical for the prophylactic effect of FH-001

Considering that adaptive immunity is not necessary for the protection, non-obese diabetes severe combined immunodeficiency (NOD-SCID) mouse was used to evaluate if the complement system or the function of natural killer cells (NKs), macrophages, or dendritic cells (DCs) is essential for the prophylactic effect of FH-001. The survival rate of 10-fold diluted FH-001-treated NOD-SCID mice was only 60% (Figure 3(C)), and the weights of these NOD-SCID mice were decreased by about 15% on the seventh day after FL/2006 challenge (Figure 3(D), Figure S3E–S3F). Considering the partial disability of NKs, macrophages, or DCs in NOD-SCID, these results suggested that innate immunity might be important for the protective effect.

Therefore, a clodronate-loaded liposome (C.L.L.) was applied to deplete the macrophages by intranasal administration every 3 days. As a negative control, liposome in PBS solution (P.L.) was used, following the same schedule. After FL/2006 challenge, P.L. + FH-001 treated BALB/c mice exhibited a 100% survival rate (Figure 3(E)) and a similar change of weight (Figure 3(E)) compared to the FH-001-treated BALB/c mice. In contrast, the total survival rate of C.L.L. + FH-001 treated BALB/c mice in three independent experiments were only 18% (Figure 3(G)), whereas the survival rate of FH-001-treated BALB/c mice was 100% (Figure 3(G)). As expected, the CD11b^low^ CD11c^+^ F4/80^+^ alveolar macrophage was dramatically decreased in the bronchoalveolar lavage fluid (BALF) from C.L.L. + FH-001 treated BALB/c mice (Figure 3(I)) when compared to the FH-001-treated BALB/c mice and negative control mice. In contrast, the proportions of CD4^+^ T cells (Figure S3G), CD8^+^ T cells (Figure S3G), B220^+^ B cells (Figure S3H), and CD11b^+^ Gr1^+^ myeloid cells (Figure S3I) in C.L.L. + FH-001 treated BALB/c mice BALFs were not decreased when compared to FH-001-treated BALB/c mice or the negative control (Figure S3G–S3I).

### Type I interferon (IFN) pathway is important for the prophylactic effect of FH-001

**Figure 4.**
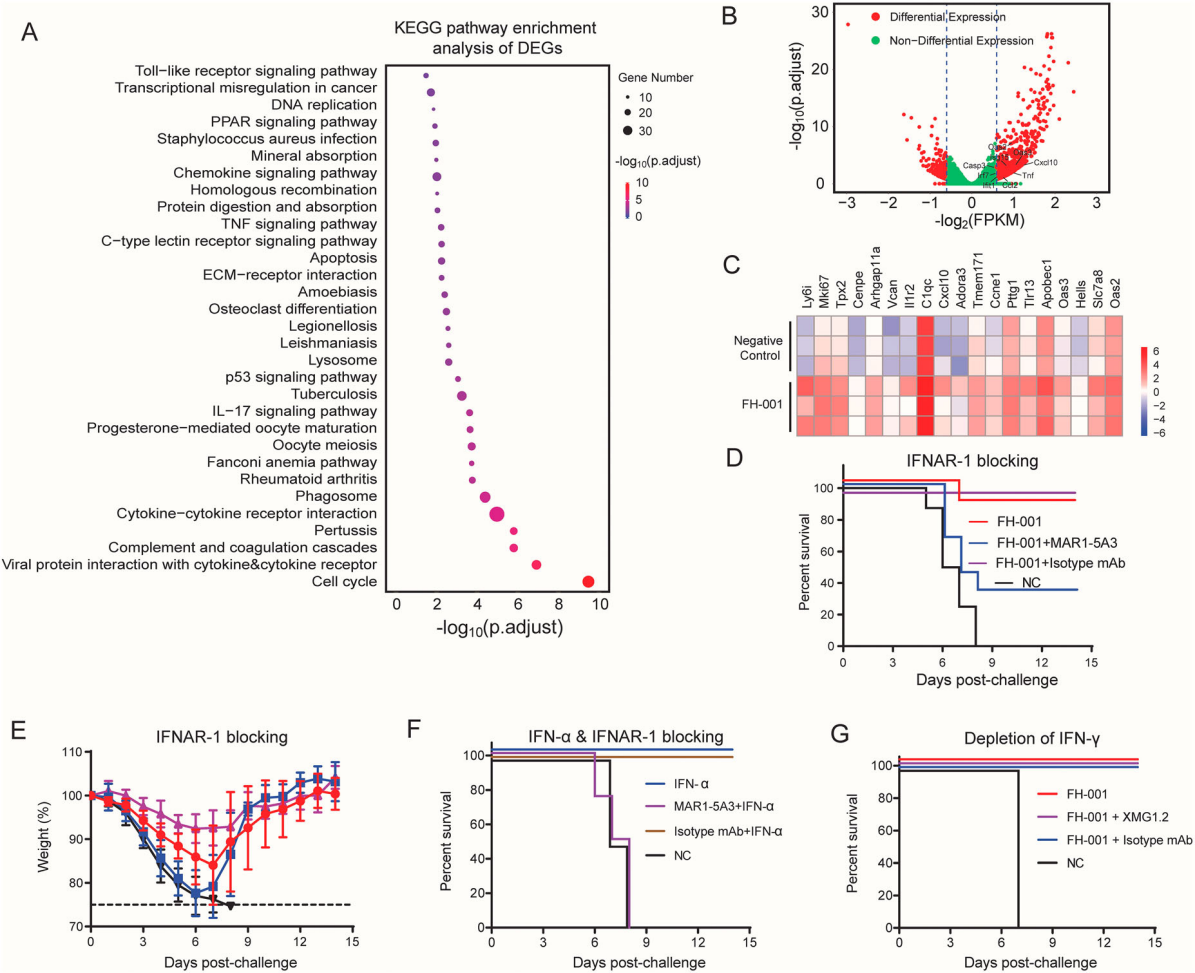
Type I interferon pathway, rather than type II interferon pathway, is essential for the protective effect of FH-001. (A) Kyoto Encyclopedia of Genes and Genomes (KEGG) enrichment of differentially expressed genes between FH-001-treated lungs and physiological saline-treated lungs from BALB/c mice. The size of the round cycle represents the enriched number of genes; the colour represents the adjusted *p*-value (p. adjust, Benjamini-Hochberg method). (B) The volcano plot shows the type I interferon pathway associated with differentially expressed genes. The values > 0 represent the genes that were upregulated in FH-001-treated mice. The dashed lines represent the position of ± 1.2-fold. (C) The heatmap shows the expression level of a part of interferon-stimulated genes in lungs from three physiological saline-treated mice (top three rows) and three FH-001-treated mice (bottom three rows). (D) Survival rates of IFNAR-1 blocking antibody (MAR1-5A3, Bio X Cell) treated mice after intranasal administration of 10-fold diluted FH-001 and lethal challenge of FL/2006. The BALB/c mice that were intranasally administered with physiological saline were challenged by FL/2006 and used as a negative control. In addition, an isotype monoclonal antibody (mAb) was also used as a control (*n* = 9 mice in FH-001 + MAR1-5A3 treated groups, *n* = 8 mice for each other groups). (E) Weight change of IFNAR-1 blocking antibody (MAR1-5A3, Bio X Cell) treated mice after intranasal administration of 10-fold diluted FH-001 and lethal challenge of FL/2006 (*n* = 9 mice in FH-001 + MAR1-5A3 treated group, *n* = 8 mice for each other groups). (F) Survival rates of recombinant interferon-α (IFN-α) treated BALB/c mice after a lethal challenge of FL/2006. The BALB/c mice that were intranasally administered with physiological saline were challenged by FL/2006 and used as a negative control (*n* = 4 mice per group). (G) Survival rates of IFN-γ neutralizing antibody (XMG1.2, Bio X Cell) treated mice after intranasal administration of 10-fold diluted FH-001 and lethal challenge of FL/2006. The BALB/c mice that were intranasally administered with physiological saline were challenged by FL/2006 and used as a negative control. In addition, a mAb with the same isotype of XMG1.2 was used as isotype control (prepared in-house), *n* = 6 mice in FH-001 + XMG1.2 treated group, *n* = 4 mice for each other groups.

To further identify specific molecular pathways associated with the prophylactic effect of FH-001, the lung homogenates from three FH-001-treated BALB/c mice and three physiological saline-treated BALB/c mice were obtained to extract the total RNA. Transcriptional analysis revealed that the TLR signalling pathway, tumour necroptosis factor (TNF) signalling pathway, and markers in the p53 signalling pathway were enriched in FH-001-treated BALB/c mice (Figure 4(A)). In addition, the transcripts of type I IFN stimulated genes (ISGs) such as *OAS2*, *OAS3*, *IFIT1*, *ISG15*, and *IRF7* were significantly upregulated in FH-001-treated BALB/c mice (Figure 4(B, C)), suggesting that the type I IFN pathway is correlated with the protective effect. Therefore, we treated BALB/c mice with a mouse IFN-α receptor 1 (IFNAR-1) blocking antibody (MAR1-5A3). As an isotype control, HPV45-specific mAb (15G11, mouse IgG1) was also included in this experiment. After the lethal challenge of FL/2006, the survival rate of MAR1-5A3 and FH-001 treated BALB/c mice (combination of two independent experiments) were dramatically decreased to 33.3% (Figure 4(D)), while the survival rate of FH-001-treated BALB/c mice or isotype control and FH-001 treated BALB/c mice were 87.5% and 100% (Figure 4(D–E)), respectively.

In addition, we directly used recombinant mouse IFN-α to test its prophylactic effect. Consistently, the IFN-α administered via a subcutaneous route did not show any protective effect (Figure S4A–S4B). However, the IFN-α intranasally treated BALB/c mice also died when lethally challenged with FL/2006 on the seventh day (Figure S4C–S4D). Further experiments revealed that one dose of IFN-α (1.5 × 10^5^ International Units) could only ensure ∼50% protection within a few days (Figure S4E), while a single dose of FH-001 could confer 100% protection at least for half a month. Further studies revealed that two dose of IFN-α (intranasally administrated at day 0, day 2 and the virus challenged at day 4) could provide 100% protection (Figure 4(F)). As expected, this prophylactic effect of IFN-α could be blocked by the IFNAR-1 specific antibody MAR1-5A3 (Figure 4(F)) but not by the isotype control. In contrast, the type II IFN pathway was not essential for the prophylactic effect of FH-001. When the rat anti-mouse IFN-gamma neutralizing antibody (XMG1.2) was used, with an isotype mAb as control, the survival rate of each group was all 100% (Figure 4(G), Figure S4F), except the negative control group (BALB/c mice pretreated with physiological saline).

### FH-001 pretreated mice could survive after challenge with IAV and respiratory syncytial virus (RSV)

Considering that the type I IFN pathway was correlated with the prophylactic effect of FH-001, as well as the non-specific antiviral ability of type I IFN, the FH-001 might have a broad-spectrum antiviral effect. Therefore, two influenza A virus strains named A/Shanghai/02/2013(H7N9) and A/California/04/2009(H1N1) and one RSV strain A2 were included in the subsequent analysis.

**Figure 5.**
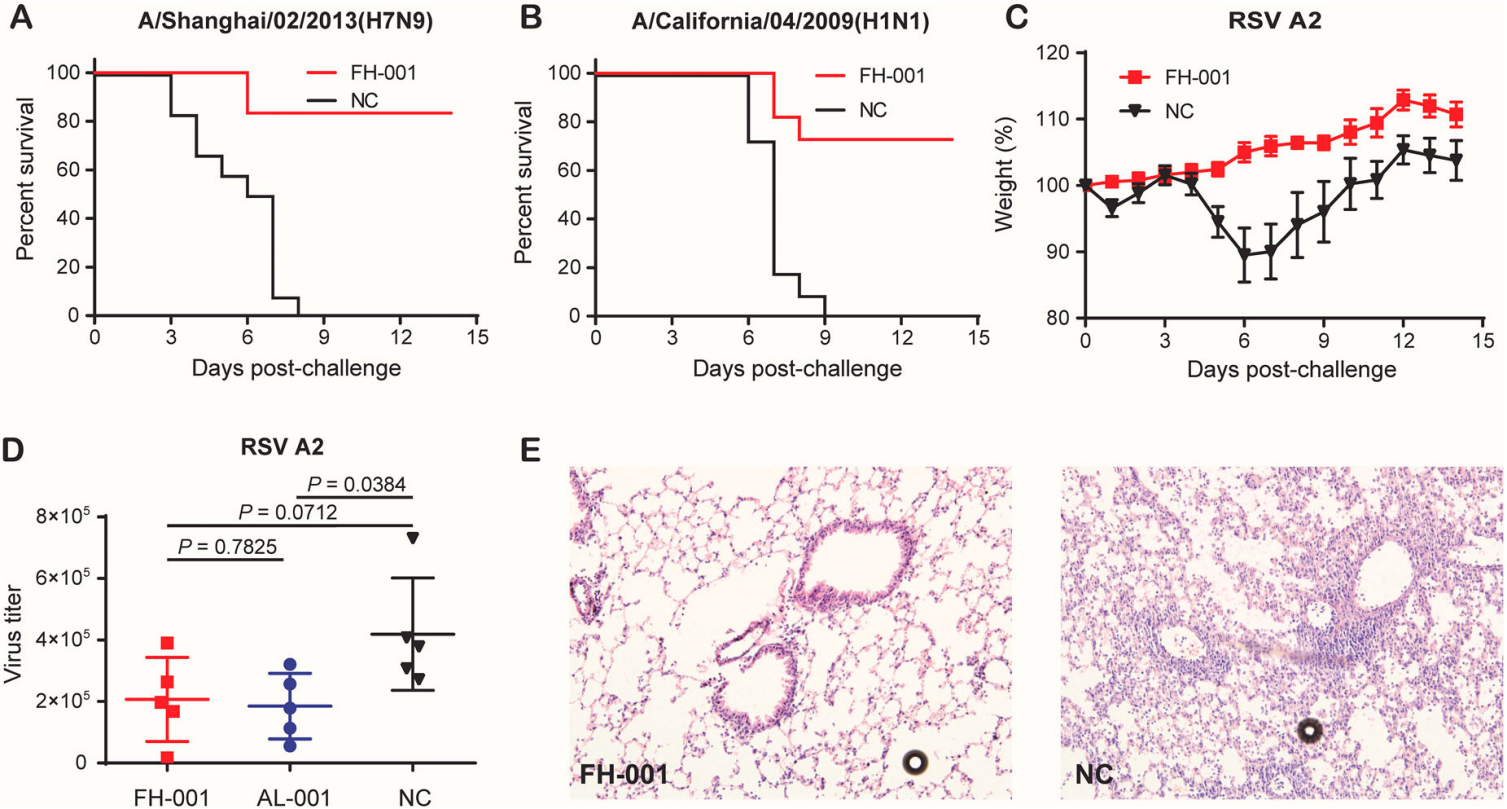
Prophylactic effect of FH-001 against influenza A virus and RSV. (A) Survival rates of FH-001-treated mice after a lethal challenge of A/Shanghai/02/2013(H7N9). The BALB/c mice that were intranasally administered with physiological saline were challenged by A/Shanghai/02/2013(H7N9) and used as a negative control, *n* = 12 mice per group. (B) Survival rates of FH-001-treated mice after a lethal challenge of A/California/04/2009(H1N1). The BALB/c mice that were intranasally administered with physiological saline were challenged by A/California/04/2009(H1N1) and used as a negative control, *n* = 12 mice per group. (C) Prophylactic effect of FH-001-treated mice after challenge by respiratory syncytial virus (RSV) strain: A2. The BALB/c mice that were intranasally administered with physiological saline were challenged by RSV A2 and used as a negative control, *n* = 10 mice per group. (D) Virus titer of RSV A2 in lungs from FH-001 or Al-001 or physiological saline-treated mice. (E) Haematoxylin-eosin staining of lung tissues from FH-001 or 0.9% saline-treated mice after challenge by RSV A2.

The survival rates of FH-001-treated BALB/c mice to A/Shanghai/02/2013(H7N9) and A/California/04/2009(H1N1) were 80% and 75% (Figure 5(A–B)), respectively; the weight decreased by nearly 20% (Figure S5A–S5B). For the RSV A2, the weight of negative control BALB/c mice was decreased by 90% on the sixth day (Figure 5(C), Figure S5C) after an intranasal challenge of the virus. In comparison, the weight of FH-001-treated BALB/c mice did not decrease after the A2 challenge (Figure 5(C), Figure S5C). Further evaluation of virus titer from mouse lungs on the fifth day after RSV A2 challenge revealed that the FH-001 or Al-001 intranasal pretreatment could promote the clearance of viruses (Figure 5(D)). Consistently, the haematoxylin and eosin staining results also showed that the lung tissues obtained from FH-001 or Al-001 pretreated BALB/c mice were normal (Figure 5(E), Figure S5D) when compared to negative control mice, suggesting that the FH-001 could provide non-specific prophylactic effect against influenza A virus and respiratory syncytial virus in the mouse model. To figure out if FH-001 or Al-001 could be used for treatment, the FH-001 or Al-001 was intranasally administered to BALB/c mice 6 h or 3 days after a lethal challenge of FL/2006. No therapeutic effect was observed (Figure S5E–S5H).

## Discussion

Diseases caused by respiratory viruses lead to a wide range of social and economic burdens every year. Seasonal influenza is still a huge health burden worldwide [33], and the ongoing COVID-19 pandemic [17,18], which emerged at the end of 2019 [3,4], has already caused over 3 million deaths until the end of April, 2021 [34]. Vaccination is the best way to prevent diseases. However, influenza virus vaccines must be updated according to the epidemic strains each year, while the protection rate and duration of COVID-19 vaccines are still unclear. In addition, the production of a new vaccine is generally time-consuming. Another way to prevent and treat respiratory disease caused by viral infection is by using antibodies, especially broad-spectrum antibodies. However, it is still difficult to screen an antibody that can recognize both influenza A viruses and influenza B viruses.

In this study, we introduced a novel way to prevent influenza viruses or RSV by activating the innate immune system via intranasal administration of adjuvant FH-001 or Al-001 alone. The FH-001 exhibited a greater protective effect than Al-001 due to the inclusion of zinc, which has an immune-enhancing effect, in FH-001. The depletion of B cells and T cells did not affect the prophylactic effect of FH-001, while the abrogation of alveolar macrophages eliminated the protective effect. Moreover, the blocking of IFNAR-1 by mAb also significantly reduced the protective effect of FH-001, indicating that type I IFN signalling is essential. Even though blocking IFNAR-1 could significantly eliminate the prophylactic effect of FH-001, no significant increase of IFN-α could be detected in the lung homogenate or serum of FH-001-treated BALB/c mice on the seventh day. This might be due to the simultaneous effect of type I IFN. This result also indicates that the intranasal treatment with FH-001 only elicited a controlled innate response since the type I IFN response returns to the normal level within few days.

Another important question is how the type I IFN is produced after the FH-001 treatment. The production of type I IFN is mainly initiated by the activation of TLRs or the cGAS-STING pathway [35–37]. However, aluminium-based adjuvants are not a specific agonist to bind TLRs. In addition, these adjuvants also do not include DNA, which could be sensed by cGAS. Therefore, we hypothesize that the activation of TLRs or the cGAS-STING pathway might be elicited by the indirect effects caused by FH-001. For example, the adjuvants might mimic the infection caused by pathogens, causing a locally controlled damage of the alveolar tissue, and leading to the release of pathogen-associated molecular patterns (PAMPs), especially the release of self-DNA in nucleus or mitochondria, which would significantly activate pattern recognition receptors (PRRs) such as TLRs. Therefore, we also tried to eliminate free-DNA by adding different concentrations of Dnase I, with various administration schedules; however, the use of Dnase I could not eliminate the prophylactic effects of FH-001. Even though these attempts do not offer positive results, further studies using *Tlr* or *Sting* knockout mice are still needed to verify whether TLRs and STING play an important role in the prophylactic effect. Except for the induction of PAMPs, another important issue is that the metal ion might directly interact with STING [38]. As reported by *Wang* et al., manganese can significantly increase the sensitivity of the cGAS-STING pathway directly [39]. Therefore, whether zinc- or aluminium-based chemical materials might also interact with the cGAS-STING pathway still needs to be investigated further. With the emergence and advantages of single-cell-based multi-omics methods, the immune and metabolic responses of the host body to the invaded pathogen can be unravelled comprehensively and quickly, especially for the outbreak of emerging diseases [5,6,40,41]. Indeed, these methods should be used further to draw an immune landscape of BALF from mice treated with FH-001.

In addition to the influenza B viruses FL/2006, the FH-001 could also protect BALB/c mice against a lethal challenge of H1N1 strain A/California/04/2009(H1N1), H7N9 strain A/Shanghai/02/2013(H7N9), and RSV strain A2. Consistently, the intranasal administration of FH-001 could also stimulate BALB/c mice to generate an effective antiviral response against the H1N1, H7N9, and RSV A2.

Finally, there are limitations to this study. For example, due to the decreased number of alveolar macrophages after the administration of FH-001, we could not obtain enough CD11b^low^ CD11c^+^ F4/80^+^ alveolar macrophages for the transfer experiment. When the alveolar macrophage is depleted but the type I IFN pathway is not blocked, FH-001 still could not elicit a sufficient protective effect. Therefore, the specific mechanism or relationship between the alveolar macrophage and the type I IFN pathway is still unclear.

In conclusion, aluminium-based adjuvant (Al-001) and an aluminium-zinc adjuvant (FH-001) can be effectively used to protect BALB/c mice against the lethal challenge of influenza B virus (B/Florida/4/2006). Further experiments revealed that the protective effect is associated with the innate immune system, especially the alveolar macrophage and type I IFN pathway, but is independent of the adaptive immunity. Due to the activation of the innate immune system, FH-001 can also protect BALB/c mice against the lethal challenge of the influenza A virus and the infection of the RSV. Therefore, we speculate that the FH-001 is likely to be used to protect host against other respiratory-related viruses. More importantly, the approach based on a rapid initiation of local immune response in the respiratory tract should have implications on the prevention and control of respiratory diseases, including the ongoing and possibly life-threatening COVID-19, which begins as a viral infection in the respiratory tract.

## Supplementary Material

Supplemental Material

## Data and materials availability

All data needed to evaluate the conclusions in the paper are present in the paper and/or the Supplementary Materials.
