# Supplementary material for: A prophylactic effect of aluminium-based adjuvants against respiratory viruses via priming local innate immunity
Source: Emerg Microbes Infect. 2022 Mar 28;11(1):914–25. doi: 10.1080/22221751.2022.2050951 (PMC8967214; doi:10.1080/22221751.2022.2050951)
Supplement: Supplemental Material [file TEMI_A_2050951_SM0080.docx]

**Supplementary Materials:**


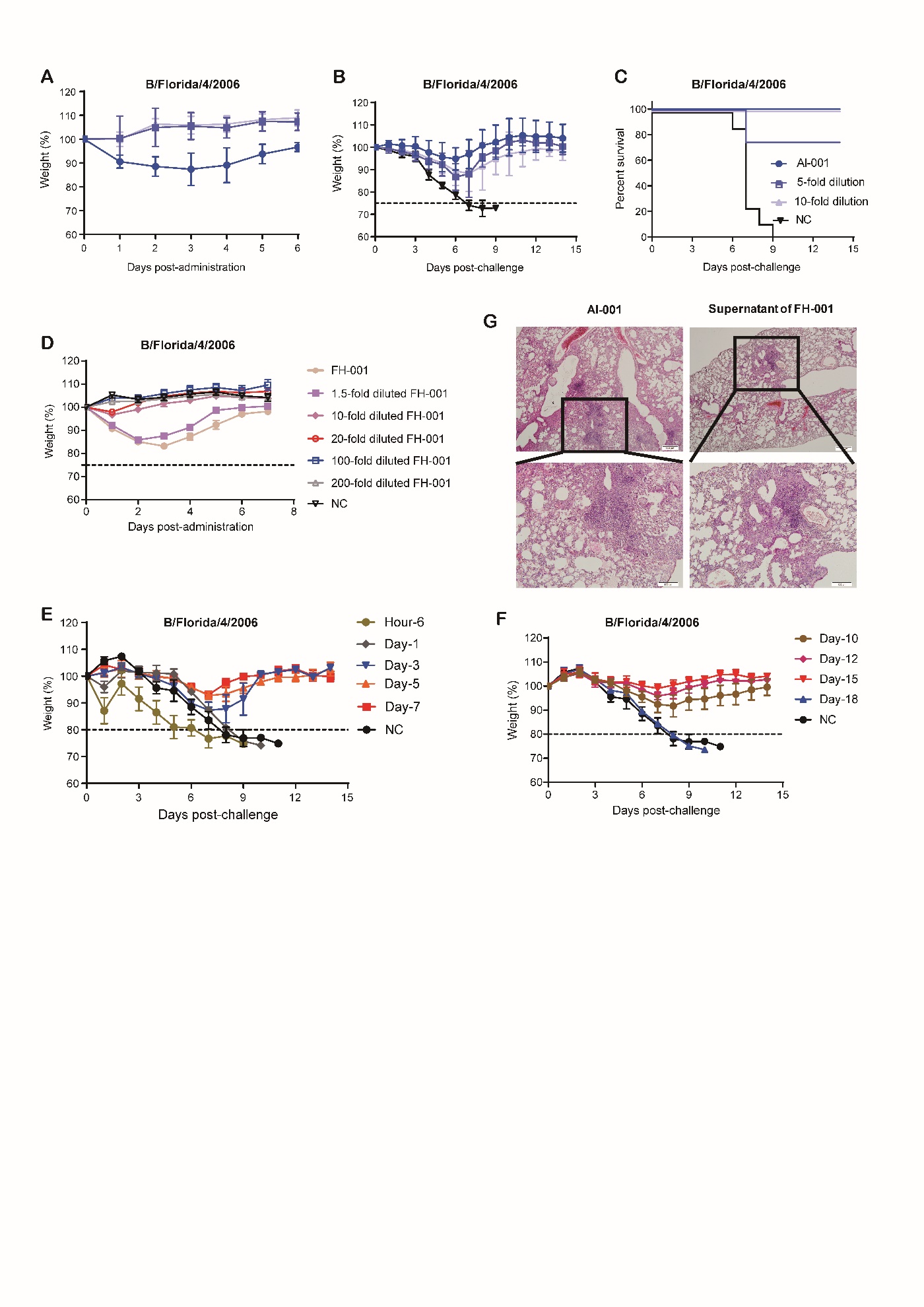


**Fig. S1. Prophylactic effect of aluminum-based adjuvants FH-001 and Al-001 (related to Figure 1).** (A) Weight change of mice after Al-001 or diluted Al-001 challenge. (B-C) Weight change and survival rate of Al-001 or diluted Al-001 treated mice after a lethal challenge by FL/2006. (D) Weight change of mice after FH-001 or diluted FH-001 challenge. (E-F) Weight change of 10-fold diluted FH-001 treated mice (treated for different times) before FL/2006 challenge. (G) Hematoxylin-eosin staining of lung tissues from Al-001 or supernatant of FH-001 treated mice after the FL/2006 challenge.


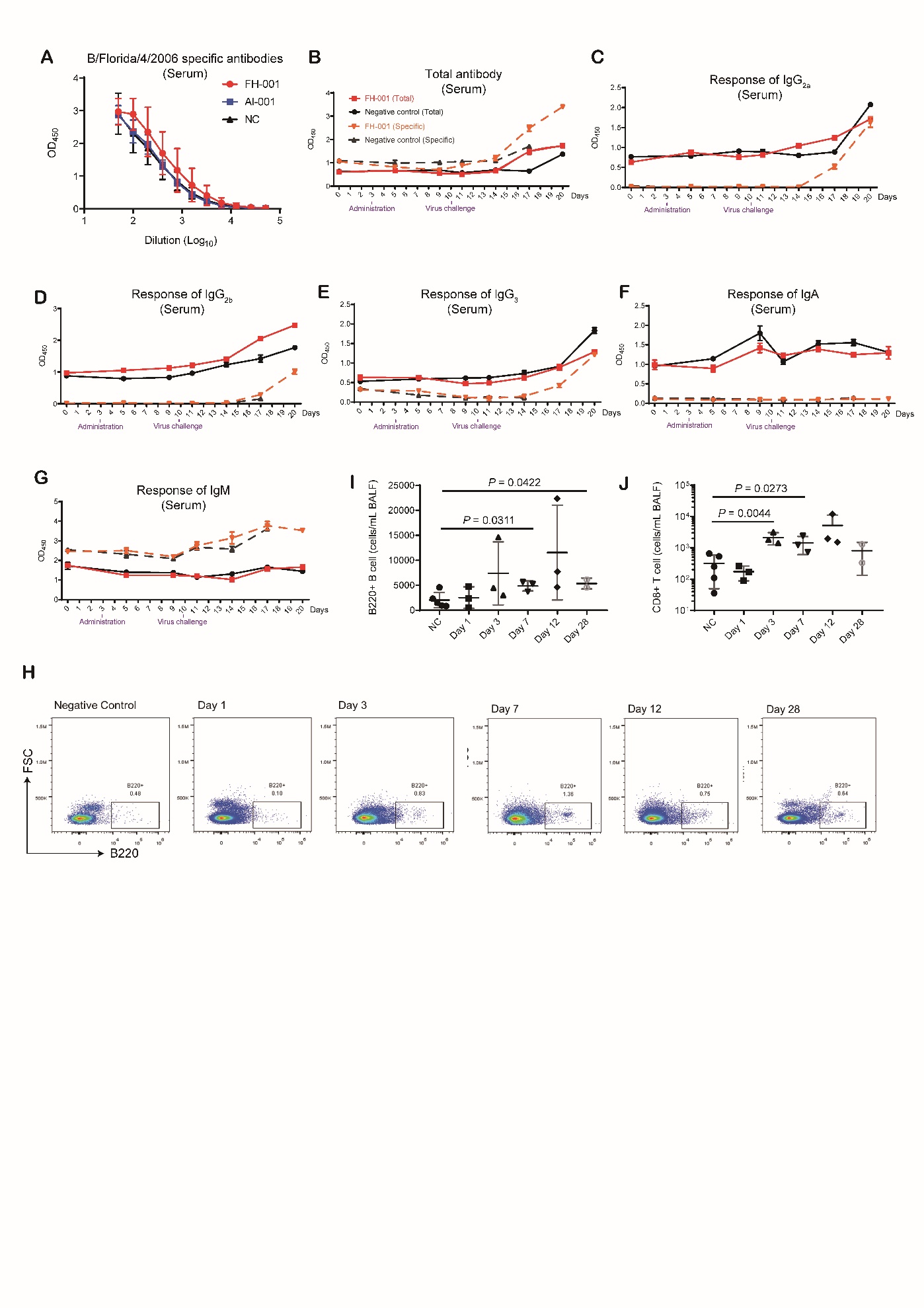
 Fig. S2. Adaptive immune response after intranasal administration of FH-001 and viral challenge (related to Figure 2). (A) Titer of FL/2006 specific antibody in FH-001 or Al-001 or physiological saline-treated mice. (B–G) Continuous total antibody titer and FL/2006 specific antibody titer in FH-001 treated and FL/2006 challenged mice. Different types of immunoglobulins were evaluated. (H) The proportion of B220^+^ B cells in mice that were treated with FH-001 for different durations. (I) Number of B220^+^ B cells in mice that were treated with FH-001 for different durations. (J) Number of CD8^+^ T cells in mice that were treated with FH-001 for different durations.


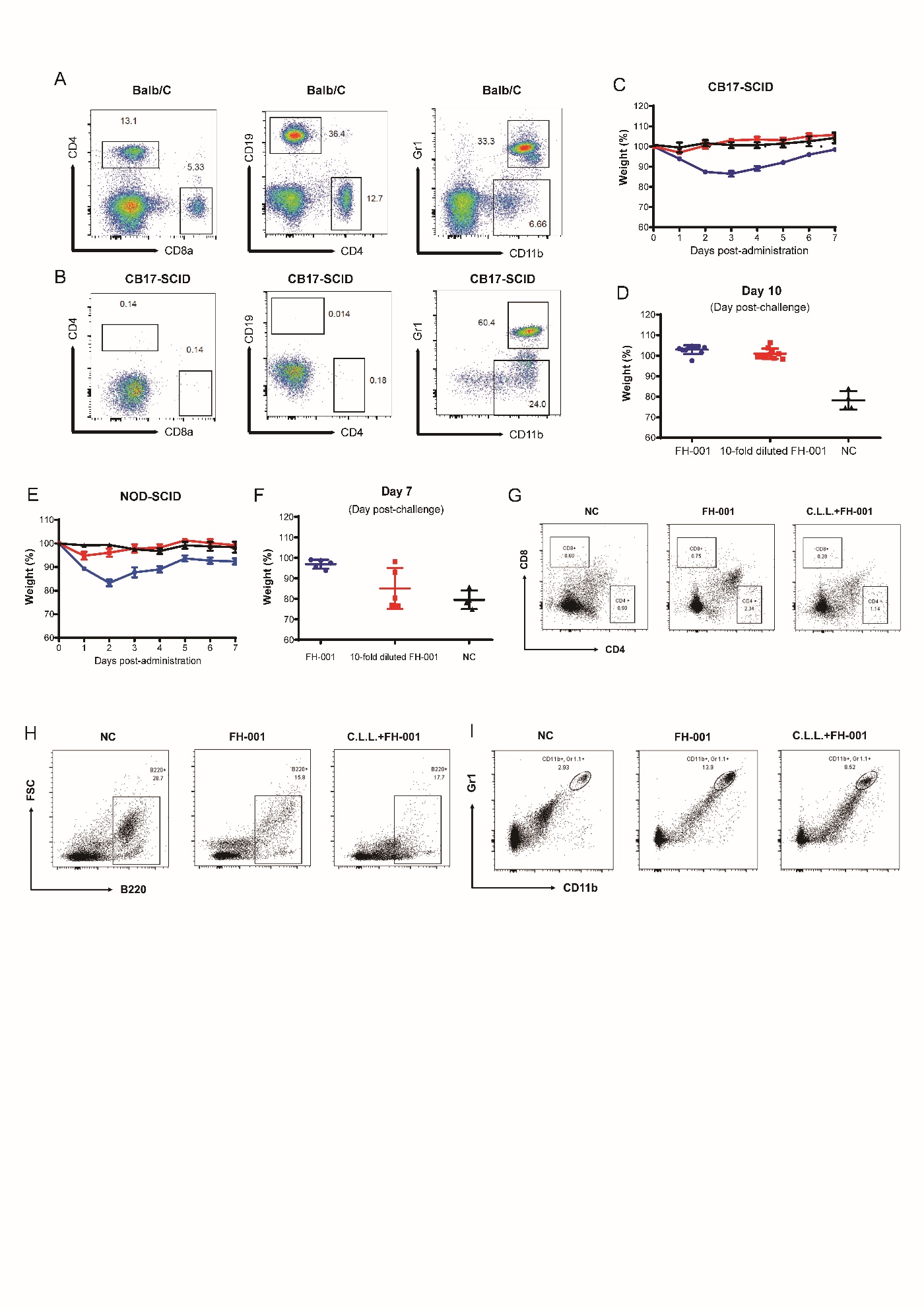


Fig. S3. B cells, T cells, and CD11b^+^ Gr1^+^ myeloid cells are dispensable for the prophylactic effect of FH-001 (related to Figure 3). (A) The proportion of CD4^+^ T cells, CD8^+^ T cells, CD19^+^ B cells, and CD11b^+^ Gr1^+^ myeloid cells in the peripheral blood of Balb/C mice. (B) Proportion of CD4^+^ T cells, CD8^+^ T cells, CD19^+^ B cells, and CD11b^+^ Gr1^+^ myeloid cells in peripheral blood of C.B-17 SCID mice. (C) Weight changes of C.B17-SCID after FH-001 or 10-fold diluted FH-001 administration intranasally. The physiological saline-treated mice were used as a negative control. (D) The weights of FH-001 or 10-fold diluted FH-001 treated C.B17-SCID on day 10 after FL/2006 challenge. (E) Weight changes of NOD-SCID mice after intranasal administration of FH-001 or 10-fold diluted FH-001. The physiological saline-treated mice were used as a negative control. (F) The weights of FH-001 or 10-fold diluted FH-001 treated NOD-SCID mice on day 7 after FL/2006 challenge. (G) Flow cytometry results of CD4^+^ T cells and CD8^+^ T cells in the BALF of physiological saline, 10-fold diluted FH-001, or 10-fold diluted FH-001 + C.L.L. treated mice. The BALF was collected on day 7 after intranasal administration of each formulation, but without viral challenge. (H) Flow cytometry results of B220^+^ B cells in the BALF of physiological saline, 10-fold diluted FH-001, or 10-fold diluted FH-001 + C.L.L. treated mice. (I) Flow cytometry results of CD11b^+^ Gr1^+^ B cells in the BALF of physiological saline, 10-fold diluted FH-001, or 10-fold diluted FH-001 + C.L.L. treated mice.


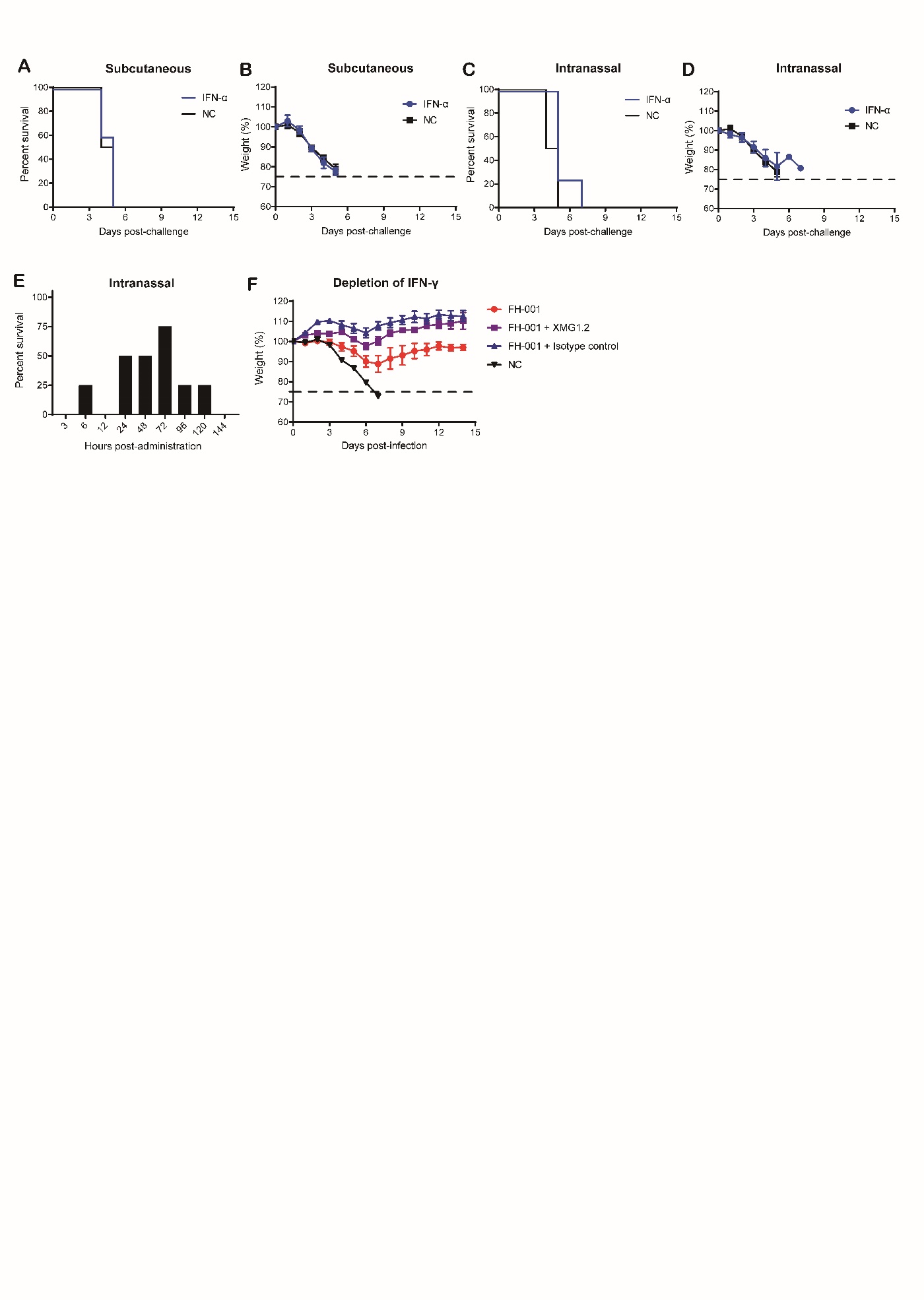


Fig. S4. Type I interferon is essential for the protective effect of FH-001 (related to Figure 4). (A-B) Survival rate and weight change of recombinant IFN-α mice (subcutaneous) after a lethal challenge of FL/2006. (C-D) Survival rate and weight change of recombinant IFN-α intranasally pre-treated mice after a lethal challenge of FL/2006. The FL/2006 was challenged on day 7 after the administration of recombinant IFN-α intranasally. (E) Percent survival of Balb/C mice after FL/2006 challenge; the mice were challenged by FL/2006 at different time points after intranasal administration of recombinant IFN-α. (F) Survival rates of IFN-γ neutralizing antibody (XMG1.2, Bio X Cell) treated mice after intranasal administration of 10-fold diluted FH-001 and lethal challenge of FL/2006. The Balb/C mice that were intranasally administered with physiological saline were challenged by FL/2006 and used as a negative control. In addition, a monoclonal antibody (mAb) with the same isotype as XMG1.2 was used as an isotype control.


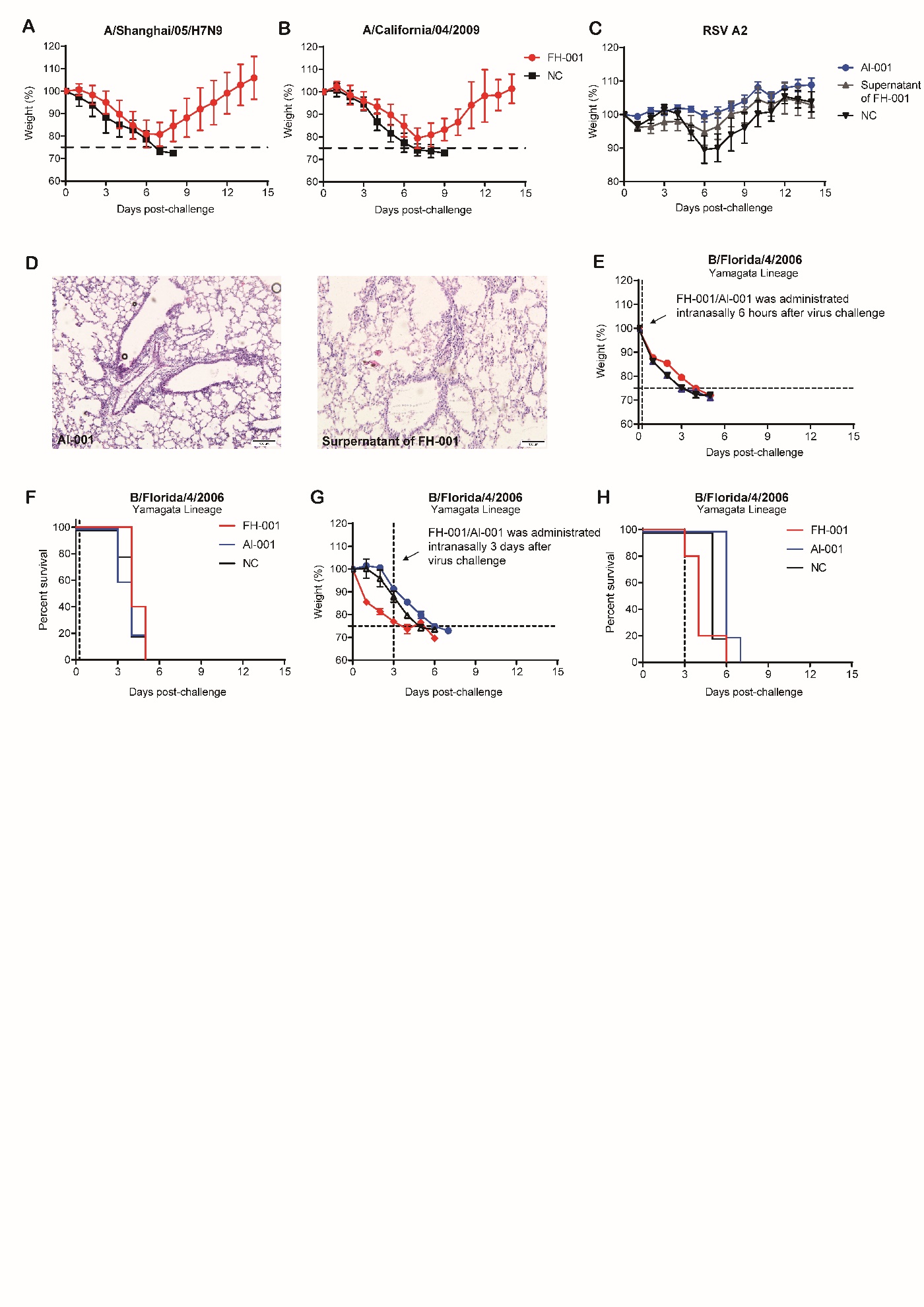


Fig. S5. FH-001 confers a prophylactic effect on mice to resist IAV and RSV (related to Figure 5). (A) Weight change of FH-001-treated mice (intranasal) after the lethal challenge of MA-A/Shanghai/05/H7N9. (B) Weight change of FH-001-treated mice (intranasal) after the lethal challenge of MA-A/California/04/2009. (C) Prophylactic effect of Al-001 or supernatant of FH-001 treated mice after challenge of RSV strain: A2. The Balb/C mice that were intranasally administered with physiological saline were challenged by RSV A2 and used as a negative control. Four mice were used in each group. (D) Hematoxylin-eosin staining of lung tissues from Al-001 or supernatant of FH-001 treated mice after the RSV A2 challenge. (E) Weight change of FL/2006 challenged mice intranasally administered with FH-001/Al-001 at 6 h after virus challenge. (F) Survival rate of FL/2006 challenged mice intranasally administered with FH-001/Al-001 at 6 h after virus challenge. (G–H) Survival rate and weight change of FL/2006 challenged mice intranasally administered with FH-001/Al-001 at day 3 after virus challenge.
